# Impact of Clinical Decision Support on Time to Order Resolution for Patients with Documented Allergies

**DOI:** 10.3390/pharmacy6030080

**Published:** 2018-08-03

**Authors:** Tenielle Watkins, Sandra M. Aguero, Michael Jaecks

**Affiliations:** Einstein Medical Center- Philadelphia, 5501 Old York Road, Philadelphia, PA 19141, USA; Saguero62@yahoo.com (S.M.A.); Jaecks01@einstein.edu (M.J.)

**Keywords:** allergy, details, antibiotics, documentation, order, verification, clinical decision support, pharmacist

## Abstract

Failure to appropriately document patient medical information, such as allergies, is an important cause of medication errors. Lack of allergy details in the electronic medical record (EMR) may prolong the pharmacist order verification process. A retrospective chart review was conducted in October 2017, to evaluate the impact of incomplete allergy details on time to antibiotic order resolution at the Einstein Medical Center Philadelphia. Details were present on 71% of orders. The difference in median time to order resolution, for orders with versus without details, was –21 min (95% CI (confidence interval), –39 to –2.9; *p* = 0.02). The difference in median time to order resolution for orders, based on pharmacist work shift was, –21 min for the first shift (95% CI, –41.2 to –0.8; *p* = 0.04), –50 min for the second shift (95% CI, –109.8 to 9.8; *p* = 0.10), and +3 min for the third shift (95% CI, –36.1 to 30.1; *p* = 0.85). Orders with an allergy intervention by a pharmacist were 2.75 times more likely (adjusted odds ratio = 2.75; 95% CI, 0.98 to 7.7; *p* = 0.06) to have a therapy change than orders without allergy interventions. Based on the results, when information about antibiotic allergies lacks details, it takes more time for pharmacists to resolve alerted orders.

## 1. Introduction

A consensus study report published by the Institute of Medicine in 2000 stated that as many as 98,000 patients die annually as a result of hospital medical errors [1]. Failure to appropriately document key patient medical information, such as allergies, is an important cause of preventable medical/medication errors [2]. A study published in the *Annals of Allergy, Asthma and Immunology* found that approximately 35.6% of patients reviewed lacked allergy information. The proper use of the electronic medical record (EMR), including important documentation regarding patient allergy history, provides medical professionals with essential information which ultimately improves clinical practice and contributes to safe and quality medical care [3].

While clinical decision support technology can be helpful in identifying interactions and alerting prescribers and pharmacists to help avoid medication errors and drug events related to allergies, a lack of appropriate documented information in the electronic medical record still poses an impediment to efficient and effective clinical decision making. 

Lack of allergy details in the EMR may prolong the order verification process, potentially resulting in treatment delays which may significantly impact patient outcomes [4,5,6]. Additionally, inaccurate or incomplete documentation may result in the generation of inappropriate clinical decision support alerts, further delaying processes. Anecdotal evidence suggested that there was insufficient detail included with documented patient allergies at the Einstein Medical Center Philadelphia (EMCP), which led us to take a closer look at the impact on pharmacy order resolution time. 

The objective of this study was to evaluate the impact of incomplete allergy details on time to pharmacist antibiotic order resolution. Allergy details were defined as documentation of the allergic reaction (e.g., hives, anaphylaxis), or documentation of previous tolerance of medications in the same class, or any combination of the two. Order resolution was defined as pharmacist verification for order approval or discontinuation. We hypothesized that a lack of allergy details would significantly prolong the order resolution process. 

## 2. Materials and Methods 

A single-center, institutional review board (IRB) exempt, retrospective chart review was completed to review antibiotic medication orders placed during the month of October 2017. Inclusion criteria comprised antibiotic orders with an associated allergy alert from 1 October–31 October, 2017. Duplicate orders and system verified orders were excluded. EMCP is a Cerner institution. The Cerner decision system identifies many combinations of drug interactions, including drug-allergy interactions, and triggers alerts accordingly to notify prescribers and pharmacists submitting and verifying medication orders. 

The primary endpoint was the difference in time to order resolution for orders with versus without associated allergy details. Secondary endpoints included: (1) differences in time to order resolution based on pharmacist shifts (first 07:00–15:00; second 15:00–23:00; and third 23:00–07:00) for orders with versus without associated allergy details; and (2), frequency of change in therapy by the ordering provider based on pharmacist allergy intervention for orders with versus without associated allergy details. An intervention is a process completed by a pharmacist when medication orders require additional clarification before they can be verified. This process includes rejecting the medication order, documenting the rejection reason with a quick summary of the information needed to move forward, and contacting the ordering provider. 

Stata version 15 (StataCorp LCC, College Station, TX, USA) data analysis and statistical software was used to analyze endpoints for statistical significance. Bivariate and multivariate statistical analyses were performed, including median regression and logistic regression. *p*-values of ≤ 0.05 were considered statistically significant.

## 3. Results

**Figure 1:**
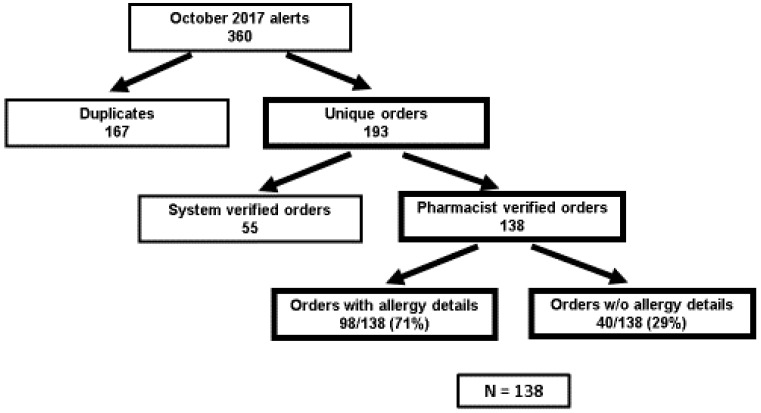
Screening results.

**Figure 2:**
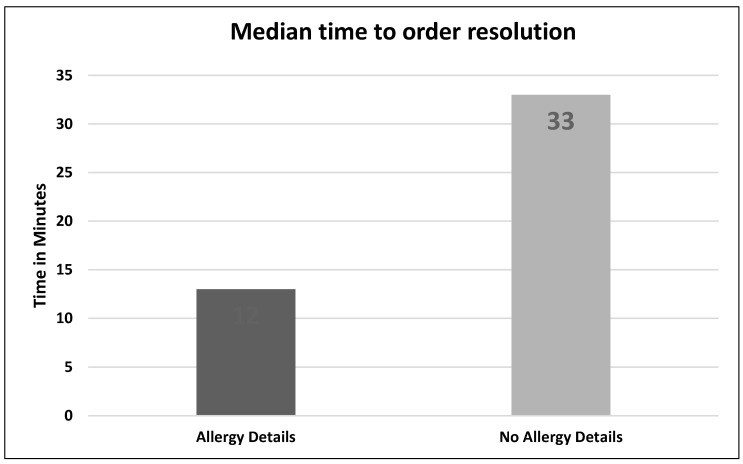
Primary endpoint results: Median time to order resolution.

**Figure 3:**
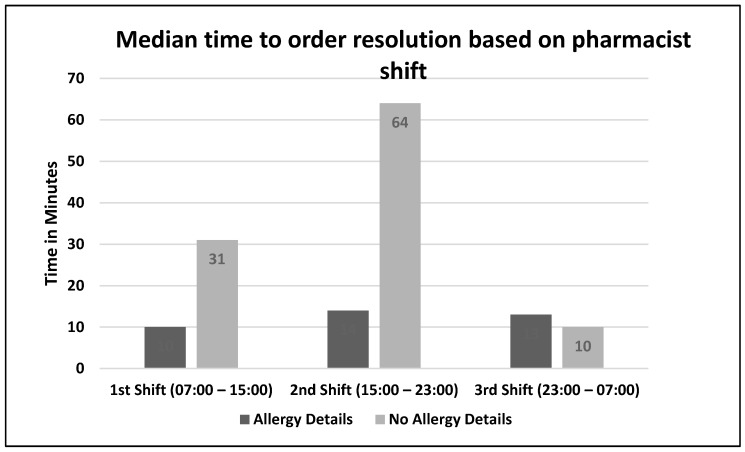
Secondary endpoint results: Median time to order resolution based on pharmacist shift.

There were 138 orders that met inclusion criteria. Allergy details were present on 98/138 (71%) of orders (Figure 1). The difference in median time to order resolution for orders with versus without details was –21 min,12 min vs. 33 min respectively (95% CI, –39 to –2.9; *p* = 0.02) (Figure 2). The difference in median time to order resolution for orders with versus without details based on pharmacist shift was –21 min for the first shift (95% CI, –41.2 to –0.8; *p* = 0.04), –50 min for the second shift (95% CI, –109.8 to 9.8; *p* = 0.10)**,** and +3 min for the third shift (95% CI, –36.1 to 30.1; *p* = 0.85) (Figure 3). Pharmacists documented allergy-specific interventions 19/98 times (19.4%) when allergy details were available and 14/40 (35%) times when allergy details were not available, and proceeded to contact ordering providers for additional clarification prior to resolving alerted orders. Of those contacts, ordering providers made a therapy change 5/19 (26.3%) times when allergy details were available, and 4/14 (28.6%) times when allergy details were not available. Therefore, when adjusted for allergy details, orders with an allergy intervention by a pharmacist were 2.75 times more likely (adjusted odds ratio = 2.75; 95% CI, 0.98 to 7.7; *p* = 0.06) to have a change in therapy than orders without allergy interventions (Table 1). A subgroup analysis was performed to analyze the median time to order resolution for the subset of orders that contained an allergy-specific intervention documented by a pharmacist. The median time to order resolution was –82 min (95% CI, –175.6 to –8.4; *p* = 0.03) for orders with allergy details, compared to those without allergy details (Table 2).

## 4. Discussion

When information about antibiotic allergies lacks details, it takes more time for pharmacists to resolve alerted orders; i.e., alerted orders with no allergy details took approximately 2.5 times longer (21 min) to resolve than those with details. We anticipated this result considering a lack of readily accessible allergy details requires a pharmacist to search through the patient’s EMR to identify previous administration and tolerance of the same or similar medications, and/or contact the ordering provider for additional details before proceeding to order resolution.

The results also identified variability in order resolution times across pharmacist shifts for orders with versus without allergy details. The longest delay in resolution time between orders with versus without details took place during the second shift (15:00 to 23:00) although this result was not statistically significant. The inpatient pharmacies were staffed appropriately according to average volume; however, 17:00 p.m. is a common departure time for a significant number of prescribers which leaves fewer prescribers available for the remainder of the evening to field phone calls and pages from the pharmacy to clarify orders, likely resulting in prolonged turnaround time. The lack of statistical significance was likely a result of the wide range, with several outliers skewing results towards resolution times > 100 min. 

The pharmacist written intervention process requires pharmacists to place other work on hold to enter a written rationale for rejecting and clarifying orders that require additional information prior to resolution. This process typically results in one or more contact attempts to the ordering provider to obtain additional information before the resolution process is completed and can take seconds to minutes depending on the level of detail supplied by the pharmacist. This likely accounts for the significant time difference of 82 min from order entry to resolution in situations where there was an allergy documented for a patient, an interacting medication was subsequently ordered, and no details were available to describe the reaction, versus those situations where allergy details were available.

There were possible limitations identified throughout the course of this study. The retrospective chart review study design resulted in the inability to interpret information beyond that which was explicitly documented in the electronic medical record. We believe this limitation was reflected in the low percentage of instances of documented pharmacy interventions upon alert of an allergy-drug order conflict. There was a lack of uniformity in pharmacist documentation of the allergy clarification process. Pharmacists at EMCP are trained to reject orders requiring additional clarification, document an appropriate intervention reason (including a thorough explanation of the rationale for rejection as well as additional information needed to resolve the order), and contact the ordering provider for additional information. For antibiotic orders that did not contain allergy details, pharmacists only provided a documented intervention/rationale for bypassing the allergy alert and proceeding with order verification 35% of the time. We strongly believe that pharmacists obtained clarification before proceeding with verifying alerted antibiotic orders more often than was documented. 

Additionally, although the study results reflected a significant difference in time to order resolution based on the availability of allergy details, there were several confounding factors identified, but not assessed, that may have also prolonged the time to order resolution. Examples include potential staffing deficits on both the pharmacy and provider sides as well as a high hospital census. 

As a result of this study, several recommendations were made to key leaders within the EMCP organization. Re-education of staff regarding the importance of appropriate, timely, and thorough allergy documentation was highly recommended. Additionally, system modifications including the implementation of required fields (such as associated allergic reaction) upon entering allergies into the electronic medical record are under consideration. Additional pharmacist education was completed with the goal of standardizing pharmacist order clarification processes.

Several considerations for potential future research were identified throughout the process of this research. Further evaluation of the clinical significance of delays in order resolution would be prudent. The results of the study reflect the significant difference in time to order resolution, but the study did not assess whether this prolonged order resolution time resulted in delays in medication administration. It is well documented that delays in the administration of medications, especially antibiotics in the setting of sepsis, may negatively impact patient outcomes; 18% of the orders included in the study were for patients who were identified as being septic. 

Our study also identified that medication orders were verified (both by pharmacists and automatically by the system in the Emergency Department) for patients with documented allergies that contained no associated allergy details. Review of these situations for administration of the medication in question, as well as subsequent allergic reaction, should be considered.

Lastly, identical research performed at a later date would provide insight as to whether the recommended changes, if implemented at EMCP, had any positive impact on time to order resolution. 

## Figures and Tables

**Table 1:** Secondary endpoint results: Changes in therapy (order discontinuation +/− alternative medication ordered) based on pharmacist intervention.

Order Type	Allergy Details	No Allergy Details
Total orders (*N* = 138)	*n* = 98	*n* = 40
Orders with allergy-specific interventions	19/98 (19.4%)	14/40 (35%)
Changes in therapy due to documented allergy	5/19 (26.3%)	4/14 (28.6%)

**Table 2:** Subgroup analysis: Median time between order entry and pharmacist resolution for orders with allergy-specific interventions.

Order Type	Allergy Details	No Allergy Details	Difference	*p*-value
All orders	12 min	33 min	–21 min	*p* = 0.02
Orders with allergy-specific interventions	11 min	93 min	–82 min	*p* = 0.03
